# Injectable Hydrogels for Localized Cancer Therapy

**DOI:** 10.3389/fchem.2019.00675

**Published:** 2019-10-11

**Authors:** Dao-yang Fan, Yun Tian, Zhong-jun Liu

**Affiliations:** Department of Orthopedics, Peking University Third Hospital, Beijing, China

**Keywords:** smart hydrogels, injectable, localized chemotherapy, stimuli responsive, drug delivery

## Abstract

Traditional intravenous chemotherapy is relative to many systemic side effects, including myelosuppression, liver or kidney dysfunction, and neurotoxicity. As an alternative method, the injectable hydrogel can efficiently avoid these problems by releasing drugs topically at the tumor site. With advantages of localized drug toxicity in the tumor site, proper injectable hydrogel as the drug delivery system has become a research hotspot. Based on different types and stages of cancer, a variety of hydrogel drug delivery systems were developed, including thermosensitive, pH-sensitive, photosensitive, and dual-sensitive hydrogel. In this review, the latest developments of these hydrogels and related drug delivery systems were summarized. In summary, our increasing knowledge of injectable hydrogel for localized cancer therapy ensures us that it is a more durable and effective approach than traditional chemotherapy. Smart release system reacting to different stimuli at different time according to the micro-environment changes in the tumor site is a promising tendency for further studies.

## Introduction

With the deterioration of the environment, the incidence of cancer is increasing year by year. In 2018, there were 18.1 million new cancer cases worldwide (9.5 million males and 8.6 million females), and the death toll was 9.6 million (5.4 million males and 4.2 million females) (Bray et al., 2018). The global cancer burden is further aggravated. One in five men and one in six women worldwide will develop cancer, and 1 in 8 men and 1 in 11 women will die for cancer (Bray et al., 2018; Sivaram et al., 2018).

Different strategies and methods are applied for cancer patients according to their different diagnose and stage. Possible therapies include chemotherapy, radiation, surgery, immunotherapy, targeted therapy, and gene therapy (Wang et al., 2013; Roy and Trinchieri, 2017; Bykov et al., 2018; Senft et al., 2018). Among those options, chemotherapy plays a pivotal role in tumor control and prevention of recurrence. The anti-tumor activity of chemotherapeutic drugs kill tumor cells with its drug toxicity (Zhou et al., 2017). Without selective or targeted killing, normal tissues could be harmed by chemical drugs along with tumor cells (Andreyev et al., 2014).

With the development of hydrogel, traditional chemotherapy drugs are booming a new life. Drugs are injected within the hydrogel directly into the tumor or adjacent to it (Elias et al., 2015; Ma et al., 2015; Pan et al., 2018). Drugs could be localized in a crosslinked 3D network of hydrophilic polymer chains (Wang et al., 2013, 2017; Bu et al., 2017). In this way, drug toxicity is limited within a localized area where tumor cells lie. Meanwhile, localized hydrogel reveals the ability for continuous efficient drug delivery in the tumor site (Yang Y. Y. et al., 2016; Yang et al., 2018). To enhance this character, a variety of systems have been developed with different composition, including polyphosphazene (PPZ), polyethylene glycol (PEG), and Polylactate glycolic acid PLGA (Bu et al., 2017; Ahmad et al., 2018; Cheng et al., 2018; Gajendiran et al., 2019).

**Figure 1:**
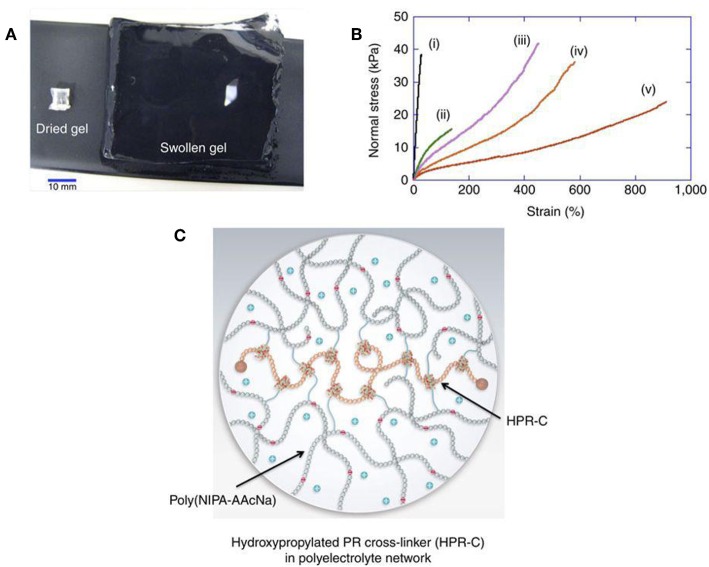
Hydrogel forms crosslinked 3D network and shows excellent water absorption capacity. **(A)** Hydrogel swelling from 129 mg (dry gel) to 80,000 mg (water-swollen gel). **(B)** Stress-strain curves of different hydrogels (i–v). **(C)** Schematic of swollen hydrogel (Bin Imran et al., 2014). Reproduced, with permission, from Bin Imran et al. (2014).

Most of the hydrogel is insoluble in water but excellent in water absorption capacity (from 10% to thousands of times their dry weight) (Bin Imran et al., 2014; Murgia et al., 2018) (Figure 1). The soft, moist surface, and affinity with the tissue greatly reduce the stimulation of the human body. Most materials that make hydrogels are non-toxic (Zhang et al., 2015; Batista et al., 2019; Gajendiran et al., 2019). According to the response to external stimuli, hydrogels can be divided into ordinary hydrogels and smart hydrogels (Gu et al., 2017). Original hydrogels are not sensitive to environmental changes (Castelletto et al., 2019; Kerdsirichairat et al., 2019; Luo et al., 2019). While smart hydrogels could be affected by pH, temperature, and photoelectricity. It results in changeable gel volume and traits for smart hydrogels (Deepa et al., 2012; Chen and Liu, 2016; Chen X. et al., 2016; Milcovich et al., 2017; Chen et al., 2019). These hydrogels are currently widely used in tissue engineering, drug delivery, and other fields (Bae et al., 2013; Del Bufalo et al., 2016; Casey et al., 2017; Castelletto et al., 2019).

## Thermosensitive Hydrogels

Temperature-sensitive hydrogels are hydrogels that change in volume as the ambient temperature changes (Klouda, 2015; Chen et al., 2018). The gel always has a certain proportion of hydrophobic and hydrophilic groups. Temperature changes can affect the hydrophobic interaction of these groups. The hydrogen bonding between the macromolecular chains causes the gel structure to change, and volume changes occur (Fan et al., 2015). The temperature at which the volume changes is referred as lower critical solution temperature (LCST) (Sapino et al., 2019). Under this temperature, the gel swells in aqueous solution. Once the temperature rises to LCST, the gel shrinks (Lei et al., 2012; Wang et al., 2015). Its unique properties can be used as a drug carrier, which is injected into the body after being combined with a drug at a low temperature (Le et al., 2018). Forming a colloidal state with the help of body temperature makes it a drug sustained-release system, which simplifies not only medical treatment but also patients' suffering (Wang et al., 2015, 2016; Yang Y. et al., 2016). Main thermosensitive injectable hydrogels include chitosan/glycerophosphate (C/GP), hyaluronic acid (HA), PLGA based hydrogel, PEG-based hydrogel, PECE, and PECT (Guo et al., 2012; Klouda, 2015; Huang et al., 2016; Le et al., 2018; Sapino et al., 2019). Their characters and drug delivery systems were summarized in Table 1.

**Table 1:** Main thermosensitive injectable hydrogel and drug delivery system.

**Hydrogel**	**Drug**	**Cell line (*in vitro*)**	**Cancer (*in vivo*)**	**Characteristics and applications**	**References**
Chitosan/glycerophosphate (CS/GP)	Indocyanine green (ICG)	Hepatocellular carcinoma (HCC)	–	Hydrogel loaded with ICG is a feasible agent for fluorescence imaging and drug delivery. It forms *in situ* compact gel and has a good ability for filling vessels	Salis et al., 2015
CS/GP	DOXorubicin (DOX)	H22 and SMMC 7721 (hepatoma)	Hepatoma	This *in situ* gelling thermosensitive hydrogel is capable of drug delivery to tumor tissue constantly and efficiently	Ren et al., 2016
Hyaluronic acid (HA) and Pluronic F127 (PF127)	DOXorubicin (DOX) and Docetaxel (DOC)	CT-26 (colorectal carcinoma)	Colorectal carcinoma	Thermosensitive hydrogels loaded with DOX and DOX has a good potential for dual drug delivery, which efficiently enhanced cancer management with minimal side effects	Sheu et al., 2016
PLGA-PEG-PLGA	PLK1shRNA/PEI-Lys and DOX	Saos-2 and MG63 (osteosarcoma)	Osteosarcoma	Localized hydrogel with RNA and DOX co-loaded is promising for efficient clinical management of osteosarcoma	Ma et al., 2014
PLGA	Paclitaxel (PTX)	M234-p (mammary tumor)	mammary tumor	Four times the efficacy of existing commercial drugs	Pesoa et al., 2018
POR–PEG–PCL	fluorescence tag	HepG2 (hepatoma)	hepatoma	Good safety and biocompatibility *in vitro* and *in vivo*	Dong et al., 2016
PCL-PTSUO-PEG	TNP/DOX/ZnPC	5637 (bladder cancer cells)	Bladder carcinoma	Double insurance from TNP/ZnPC and TNP/DOX loaded in hydrogel for the management of bladder carcinoma	Huang et al., 2018
PEG–PCL–PEG, PECE	PTX	4T1 (breast cancer)	Breast cancer	Great antitumor effect and wound healing promotion.	Lei et al., 2012
PECT	PTX	MCF-7	Breast cancer	High concentration in tumor tissue for 21 days	Lin et al., 2016

**Figure 2:**
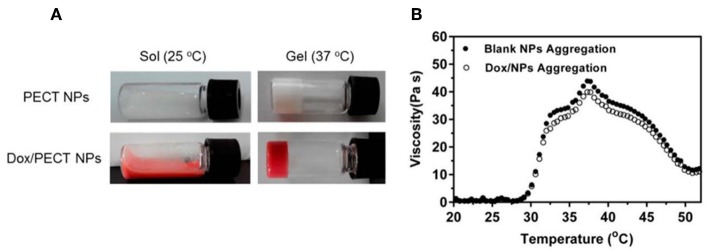
The thermosensitive property of hydrogel. **(A)** State of the thermosensitive hydrogel at different temperatures (25°C, 37°C). **(B)** Hydrogel viscosity increase of aqueous solution as a function of temperature. Source: Reproduced, with permission, from Huang et al. (2016).

In one study (Huang et al., 2016), injectable thermosensitive doxorubicin (DOX) delivery system was developed with PECT hydrogel. Instead of hydrogel based on free DOX diffusion, which suffered from rapid drug clearance and poor drug penetration in tumor tissue, self-polymerized drug-loaded nanoparticles were encapsulated into PECT hydrogel (Huang et al., 2016). After *in vivo* injection, PECT gel exhibited a transition phase between sol and gel. The viscosity increased abruptly once the temperature is higher than 28°C. With the highest viscosity at 37°C, the hydrogel turned to gel from sol (Figure 2). Loaded nanoparticles were dissociated from hydrogel and diffused within tumor tissue by EPR effect. Intracellular chemical drug release limited its toxic effects and enhanced its anti-tumor effectiveness. Contrasted with intravenous drug injections (*I.V*.), a thermosensitive hydrogel with nanomedicine loaded is an efficient drug delivery system, which enabled continuous drug release around tumor tissues (Huang et al., 2016).

**Figure 3:**
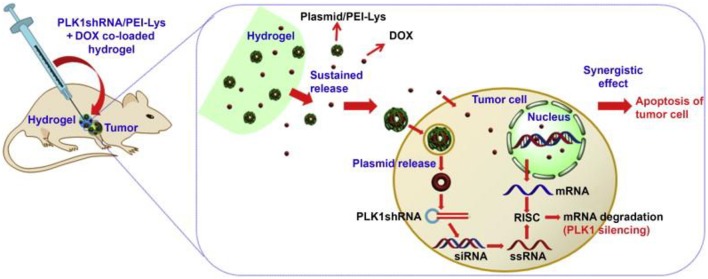
Schematic for synergism of PLK1shRNA/PEI-Lys and DOX from the hydrogel promotes tumor apoptosis on osteosarcoma in nude mice. Reproduced, with permission, from Ma et al. (2014).

Two or more elements loaded in one thermosensitive hydrogel has emerged as a promising drug delivery system for its superior anti-tumor efficacy. Polo-like kinase 1 (PLK1) gene is recognized as a key regulator of tumor cell meiosis and mitosis (Ma et al., 2014). RNA interference-based on PLK1shRNA can specifically reduce the function of the target gene in the tumor. A strategy of DOX and PLK1shRNA/PEI-Lys co-delivery hydrogel was developed for the treatment of osteosarcoma (Ma et al., 2014). In this method, PLK1shRNA/PEI-Lys in the hydrogel can greatly enhance the anti-tumor effect of DOX. With the synergistic effect from PLK1shRNA/PEI-Lys and DOX, significant osteosarcoma apoptosis was caused by the co-loaded hydrogel (Figure 3) (Ma et al., 2014).

## pH-Sensitive Hydrogels

Glycolysis of tumor cells can cause acidification in the environment next to tumor tissues, resulting in lower pH value in the extracellular matrix than normal tissues (Kenney et al., 2018; Hu et al., 2019). A pH-sensitive hydrogel is a polymer gel in which the volume of the hydrogel changes depending on the pH of the external environment and the ionic strength (Liao et al., 2017; Liu et al., 2019). Such gels contain a large number of readily hydrolyzable or protonated acids, base groups such as carboxyl groups and amino groups (Lym et al., 2016). The dissociation of these groups is affected by the external pH. When the external pH changes, the degree of dissociation of these groups changes correspondingly, causing the internal and external ion concentration to change (Norouzi et al., 2016). In addition, the dissociation of these groups will destroy the corresponding hydrogen in the gel. The bond reduces the cross-linking point of the gel network, causing a change in the structure of the gel network and the degree of swelling of the hydrogel (Qu et al., 2017; Oroojalian et al., 2018). With this property, the rate of diffusion and release of the drug in the gel can be conveniently adjusted and controlled (Samanta et al., 2015).

**Figure 4:**
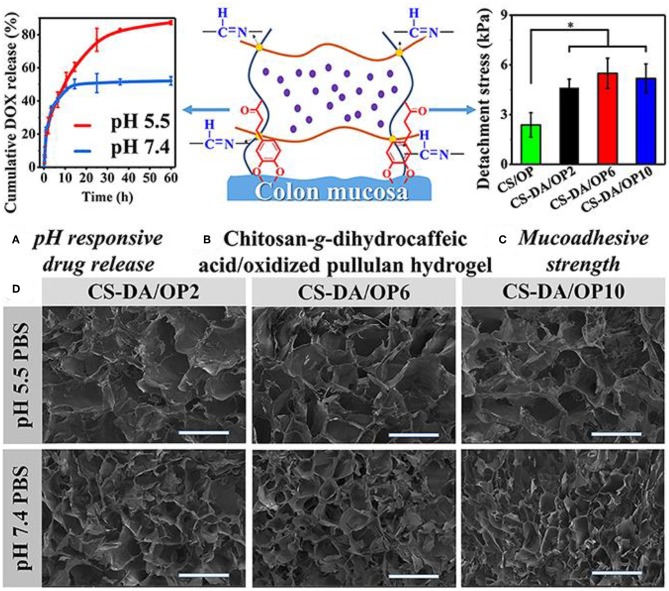
Characters of the pH-sensitive hydrogel. **(A)** pH-responsive drug release. **(B)** Schematic for pH-sensitive hydrogel. **(C)** Mucoadhesive strength of different elements. **(D)** SEM images of the pH-sensitive hydrogel after swelling in PBS with different pH values. *P < 0.05. Reproduced, with permission, from Liang et al. (2019).

A variety of elements for pH-sensitive hydrogel were explored in the past decades. Their characters and drug delivery systems were summarized in Table 2. One of the choices is based on chitosan-grafted-dihydrocaffeic acid (CS-DA) and oxidized pullulan (OP) (Liang et al., 2019). With classical drug for anti-tumor therapy, DOX-loaded hydrogel was tested to explore its reactions for the pH changes in the tumor environment. With glycolysis in the tumor site, a decrease of pH triggered the drug release (Liang et al., 2019). Compared with the morphology of hydrogel at pH 7.4, significant disintegration of hydrogel resulted in larger pore size at pH 5.5 (Figure 4) (Liang et al., 2019). After 60 h at pH 5.5, more than 80% of DOX was released from the hydrogel. The hydrogel was co-cultured with Hct116 cells (colon tumor cells) to test its anti-tumor effect (Liang et al., 2019). DOX is continuously stable released from hydrogel at pH 5.5 and 7.4. In both conditions, DOX can be effectively released for more than 3 days (Liang et al., 2019).

**Table 2:** pH-sensitive hydrogels and drug delivery system.

**Hydrogel**	**Drug**	**Cell line (*in vitro*)**	**Cancer (*in vivo*)**	**Characteristics and applications**	**References**
Acrylic Acid and PEGDA	Salicylic Acid (SA)	3t3 fibroblast cells	–	Sa based pH sensitive hydrogel reveals good pH sensibility and great biocompatibility	Demirdirek and Uhrich, 2017
CS and GP	DOX	Mcf-7 cells (breast cancer)	Breast cancer	With an LCST of 39°C, the gel reveals DOX when pH = 5.5	Fathi et al., 2019
Chitosan Dihydrocaffeic Acid (CS-DA) and Oxidized Pullulan (OP)	DOX and Amoxicillin	Hct116 cells (colon tumor cells) and e. Coli	Colon tumor and infection	DOX and amoxicillin co-loaded hydrogel response to pH decrease. The hydrogel is an ideal system for mucosa-localized tumor and infection management	Liang et al., 2019
GC-PF127	DOX	H22 (breast cancer)	Breast cancer	Response to small pH change, hydrogel release DOX-loaded micelles with tumor-targeted	Liu et al., 2017
CEC-PEGDA	DOX	Hepg2 (liver cancer) and l929	Liver carcinoma	Good cytocompatibility and anti-tumor effect. The pH-responsive hydrogel is a promising delivery system for liver cancer treatment	Qu et al., 2017
PEGMA and AAC	5-FU	Hepg2 (liver cancer) and LO2	Liver carcinoma	Controlled and targeted drug delivery for liver carcinoma	Yue et al., 2019
CS/HA/GP	DOX	Hela (cervical cancer)	Cervical cancer	Good pH sensitivity for the localized management of cervical cancer	Zhang et al., 2018

In recent years, aspirin has been found to inhibit carbon monoxide synthase, inhibit nitrite-mediated DNA damage, reduce surviving, inhibit nuclear transcription factors, proteasomes, and calcium-activated neutral proteasome genes by inhibiting cyclooxygenase (Lu et al., 2008; Choi et al., 2013). The expression and other mechanisms play an anti-tumor effect. After loading aspirin into the hemicellulose hydrogel, it was found that 85% of the drug could be released continuously for 5–6 h under pH 7.4 (Choi et al., 2013). Sun et al. successfully prepared a series of acrylic acid and acrylamide copolymer grafted hemicellulose hydrogels by free radical polymerization (Sun et al., 2013). The combination of aspirin and the drug showed that the release rate of the drug in the simulated gastric juice was slower, and the release rate in the simulated intestinal fluid was significantly faster than that of the simulated gastric juice. When the release time was 12 h, the cumulative release rate reached 90%, which shows excellent sustained release properties (Sun et al., 2013).

In addition, Wang et al. first inserted dexamethasone phosphate into molecularly imprinted polymer nanospheres and loaded the polymer onto a pH-sensitive hydrogel, making it a biosensor that inhibits inflammation (Wang et al., 2010). Since the inflammatory reaction can lead to an acidic environment, this pH-sensitive hydrogel can rapidly release the drug at pH 6.0~7.4 to inhibit the inflammation (Wang et al., 2010). This different method is resistant to pH-sensitive hydrogels. The application of oncology drugs has brought new ideas.

## Photosensitive Hydrogels

The mechanism of a light-sensitive hydrogel is divided into two types according to the properties of the photosensitive material (Chang et al., 2019). One is to directly add the photosensitive molecular material to the temperature-sensitive gel, and convert the light energy into heat energy to make the temperature inside the gel reach the phase transition temperature. In this way, hydrogel produces photosensitivity. Another kind of chromophore is introduced into the gel structure (Norouzi et al., 2016). The physicochemical properties of the chromophore are changed when subjected to light stimulation. By changing the network structure, hydrogel macroscopically exhibits photosensitivity. Usually, a structure such as azobenzene, spiropyran, o-naphthoquinone, anthracene, nitrophenyl, and coumarin is introduced into the gel (Tam et al., 2017). Among them, ruthenium, nitrophenyl, and coumarin compounds mainly take advantage of photo-cleavage photosensitive groups, which are bonded to the hydrophobic end through an aryl methyl bond (Norouzi et al., 2016; Tam et al., 2017). Under ultraviolet light or near-infrared light, the ester group is broken. The photosensitive reaction is caused. The hydrophobic end is converted to a hydrophilic end, causing the gel to dissociate. The azobenzene compound is controlled by the conversion of the cis-trans structure. Their characters and drug delivery systems were summarized in Table 3.

**Table 3:** Photosensitive hydrogels and drug delivery system.

**Hydrogel**	**Drug**	**Cell line (*in vitro*)**	**Cancer (*in vivo*)**	**Characteristics and applications**	**References**
Hyaluronic Acid (HA)	Matrix metalloproteinase (MMP)	MDA-MB-231 cells (breast cancer)	–	Biomimetic 3D cell culture model for cancer researches.	Tam et al., 2017
DNA Polyacrylamide Conjugate (DPC)	DNA and DOX	CEM (lymphocytic leukemia)	Lymphocytic leukemia	Controlled release with DNA crosslinked inside the photo-responsive hydrogel	Kang et al., 2011
Hyperbranched polyprodrug (PPM)	PPM	A549 cells (lung cancer)	Lung Cancer	DOX and amoxicillin co-loaded hydrogel response to pH decrease. The hydrogel is an ideal system for mucosa-localized tumor and infection management	Guo et al., 2018

**Figure 5:**
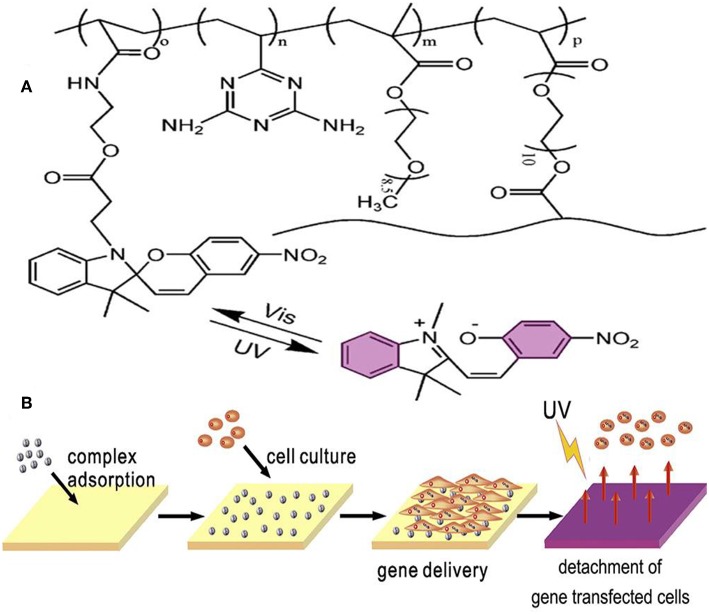
Schematic structure and mechanism of the photosensitive hydrogel. **(A)** Schematic molecular structure of hydrogel. **(B)** UV irradiation triggers the detachment of cells from the surface of the hydrogel. Reproduced, with permission, from Wang et al. (2014).

One of the applications for photosensitive hydrogels is the platform for cell culture and 3D tumor micro-environment studies. With advantages of its photosensitive character, cell detachment on the surface of hydrogel was done layer-by-layer to form a 3D cell culture medium (Figure 5) (Wang et al., 2014). Photoinitiated copolymerization of P (OEGMA-co-VDT-co-SPAA) (POVSP) hydrogels happened with UV irradiation. The compressive strengths of hydrogels were up to 5.1 MPa, which is strong enough for cell culture (Wang et al., 2014). It is revealed that photosensitive hydrogel is suitable for 3D cell culture model, which is vital for the study of the mechanism for tumor development.

**Figure 6:**
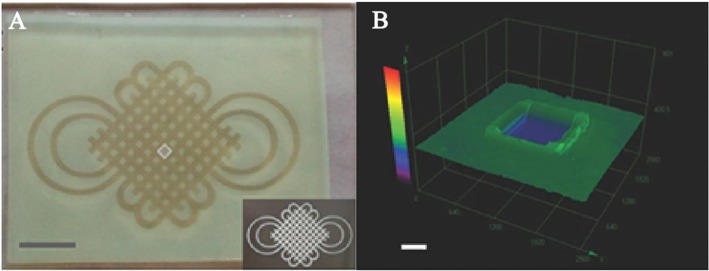
Photoactive self-shaping hydrogel spontaneous swelling caused by UV irradiation. **(A)** Hydrogel shaped by selected UV irradiation to form a designed pattern. **(B)** 3D image of a square unit for the design of UV irradiation. Reproduced, with permission, from Liao et al. (2015).

To achieve the same purpose, a photocleavable terpolymer hydrogel was developed as the basic technique for 3D bio-printing. This hydrogel is capable of self-shaping directly to the UV irradiation. It is designable by using selective illumination to UV light with the specific area covered with darkness (Figure 6) (Liao et al., 2015). The printable hydrogel is an inspiring design for controlled drug delivery with district distribution. It is a key technique for the realization of 4D drug delivery with both dimensions of time and space. With drugs loaded in the 3D space of hydrogels, dynamic drug release can be realized. In this process, different drug could be controlled to be released in different time with purpose (Xu et al., 2015; Chen Y. et al., 2016; Kim et al., 2017; Guo et al., 2018). This is the typical way of 4D drug delivery with additional dimensions of time.

## Dual-Sensitive Hydrogels

With the increasing requirements for the precision of controlled release of drugs, multi-sensitive hydrogels have received more and more attention (Bardajee et al., 2017). In particular, co-sensitive hydrogels for temperature and pH is widely researched. Temperature and pH are two important factors in physiological, biological, and chemical systems (Bardajee et al., 2017; Fathi et al., 2019). The temperature-pH double-sensitive hydrogel consists of a temperature-sensitive and pH-sensitive two-part hydrophilic polymer network (Lym et al., 2016). Usually formed with two or more monomers or polymers, which respond to temperature and pH, respectively.

**Figure 7:**
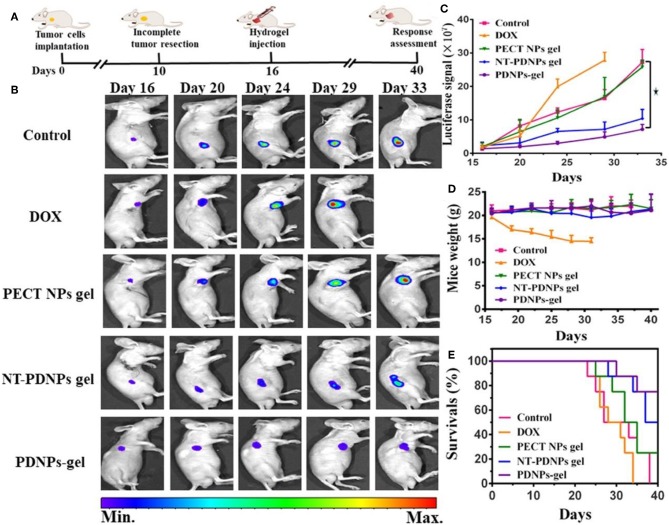
*In vivo* luciferase-tagged tumor model with the management of hydrogel. **(A)** Experiment process. **(B)** Bioluminescence images of mice treated with different formulations, **(C)** Quantified bioluminescence for tumors in mice. **(D)** Mice weight changes. **(E)** Survival rates of mice treated with different methods. Reproduced, with permission, from Liu et al. (2019).

The combination of temperature and pH sensitivity is crucial for the management of locoregional tumor recurrence (Mackiewicz et al., 2019). A novel pH-sensitive thermosensitive hydrogel loaded with modified doxorubicin-based prodrug nanoparticles (PDNPs), which is more efficient for tumor management than free DOX (Liu et al., 2019). Good biocompatibility and anti-tumor activity were verified by *in vitro* uptake and cell toxicity. For *in vivo* experiment, 4T1 cells with luciferase-tagged expression were implanted into mice. Management by temperature and pH co-sensitive hydrogel was remarkable (Figure 7) (Liu et al., 2019). It is a promising strategy for preventing the locoregional recurrence of the tumor.

Co-sensitive hydrogel with dual photoluminescence was developed with PL and PNIPAM (Zhao et al., 2016). This hydrogel contains a core which was made up of a red-emission complex and a blue-emission d-TPE. This nanoparticle is sensitive to the change of temperature and pH (Zhao et al., 2016). This hydrogel is stimulated by both temperature and pH and is more adaptable to the complex environment of human body fluids. In addition, the application of two or more materials, through their interaction, not only can improve the mechanical strength of the hydrogel, but also improve the precision of controlled release. With this character, the stimuli-responsive hydrogel has a wide application in medical imaging, cancer diagnosis, and advanced antitumor drug delivery (Zhao et al., 2016).

## Conclusions

The unique character of hydrogel makes it an efficient functional medium for drug delivery (Zhang et al., 2018; Fathi et al., 2019; Mackiewicz et al., 2019). Given the limits from chemical drug toxicity for normal tissue and organs, localized drug delivery system by hydrogel has been a crucial method for cancer management. Related studies mainly focused on the delivery function and the methods of stimuli-response (Lym et al., 2016; Bardajee et al., 2017; Wei et al., 2017; Fathi et al., 2019). The smart hydrogel was developed with accurate responses to tiny changes in temperature, pH, and light. For now, drugs can be easily delivered to cancer tissue at the right time point. In the future, co-loaded drugs, including DNA, RNA, protein, and related products, would be a key point. The constantly accurate drug delivery system can realize anti-tumor drugs release followed by tissue repair factors. In this way, demission of time and space for drug delivery would be mixed in one hydrogel, making it a 4D functional hydrogel. It can make hydrogel a perfect choice for local chemotherapy and cancer management.

## Author Contributions

All authors listed have made a substantial, direct and intellectual contribution to the work, and approved it for publication.

### Conflict of Interest

The authors declare that the research was conducted in the absence of any commercial or financial relationships that could be construed as a potential conflict of interest.
